# Does lifelong learning matter for the subjective wellbeing of the elderly? A machine learning analysis on Singapore data

**DOI:** 10.1371/journal.pone.0303478

**Published:** 2024-06-05

**Authors:** Zheng Fang, Nicholas Sim

**Affiliations:** 1 Graduate Studies, Singapore University of Social Sciences, Singapore, Singapore; 2 School of Business, Singapore University of Social Sciences, Singapore, Singapore; Federation University Australia, AUSTRALIA

## Abstract

Our study explores whether lifelong learning is associated with the subjective wellbeing among the elderly in Singapore. Through a primary survey of 300 individuals aged 65 and above, we develop a novel index to capture three different aspects of subjective wellbeing, which we term “Quality of Life”, “Satisfaction with Life” and “Psychological Wellbeing”. Utilizing both supervised and unsupervised machine learning techniques, our findings reveal that attitudes towards lifelong learning are positively associated with quality of life, while participation in class activities is positively associated with all three measures of wellbeing. Although the study does not establish causality, it highlights a connection between lifelong learning and the perceived wellbeing of the elderly, offering support for policies that encourage lifelong learning among this population.

## 1. Introduction

One of the major challenges facing advanced economies is the rapidly aging population. Singapore, with one of the fastest rates of aging in the world, is no exception. By 2030, approximately one third of Singapore’s residents will be 65 and above, and by 2050, that number is projected to rise to half of the population [1]. The aging process brings with it a host of issues such as loneliness and decreased independence, which can eventually lead to self-harm. For instance, elderly suicide has been a prevalent problem in Europe and North America; Singapore, despite its close-knit communities, is not immune to these challenges. According to the Samaritans of Singapore (SOS), the year 2020 saw the highest number of suicides among the elderly (aged 60 and above) in the last 30 years in Singapore.

As society ages, it is important to develop an ecosystem of early intervention and support for healthy aging, particularly the mental and emotional health of the elderly. The aging population will lead to sharp increases in the cost of healthcare, which raises the tax burden on the young. Promoting the “health-span” of seniors and their mental wellbeing will be crucial from the policy perspective and for long-term sustainability of public services.

How do we, then, promote active aging? In this paper, we explore the association between lifelong learning and the wellbeing of older adults. Lifelong learning is the continuous enhancement of skills and knowledge throughout one’s entire lifetime, beyond formal education [2]. In Singapore, it has been promoted, as part of its $3 billion Action Plan for Successful Ageing (APSA) launched in 2015, to encourage active aging. Research on other countries has shown that lifelong learning can help to maintain cognitive functions and delay the onset of age-related health problems such as dementia, amnesia, and depression [3]. As technologies continue to evolve at a rapid pace, keeping up with learning has become even more crucial for seniors, as failure to adapt may cause them to feel left behind [4].

Our study is motivated by recent policies that promote lifelong learning for successful aging and aims to provide new empirical insights into the potential association between lifelong learning and subjective wellbeing among older adults in Singapore. We utilize machine learning techniques on primary data we collected on the Singapore elderly population to achieve this objective. Our primary data are based on a survey on 300 senior residents aged 65 and above. We collect information about their background, engagement in social activities, attitudes towards lifelong learning, and responses related to different aspects of subjective wellbeing.

For our empirical analysis, we draw from the index number literature to develop a novel index each for three different aspects of subjective wellbeing, which are termed “Quality of Life”, “Satisfaction with Life” and “Psychological Wellbeing”. We then employ a combination of supervised and unsupervised machine learning techniques to explore their associative relationship with measures of lifelong learning (if any). For unsupervised learning, we introduce the use of multiple correspondence analysis (MCA) to study the association between wellbeing and lifelong learning. MCA is a dimensionality reduction factor technique, similar to the more popular principal component analysis (PCA), but designed for nominal or ordinal categorical data, which our survey responses are. For supervised learning, we construct decision trees to identify if lifelong learning is an important predictor of wellbeing and complement these results with statistical inference from regressions. In the context of our study, the decision tree will use the attributes of the individuals to group in a way that ensures that these individuals are as homogeneous as possible in their levels of well-being. The use of machine learning, such as decision trees, has become increasingly popular in the field of bioinformatics and interdisciplinary studies. Examples such as DP-BINDER [5], iMethyl-STTNC [6], AFP-CMBPred [7], and iAFPs-EnC-GA [8] have demonstrated the power and versatility of machine learning in unraveling complex biological patterns. By aligning our approach with these advancements, we aim to contribute to the growing body of knowledge that underscores the significance of machine learning in social science studies.

Our MCA and decision tree analyses suggest a positive association between attitudes towards lifelong learning and measures of wellbeing reflecting the quality of life and life satisfaction, but not with psychological wellbeing. The regression results further demonstrate that attitudes towards lifelong learning are statistically significant for quality of life only, indicating that lifelong learning attitudes matter for certain aspects of wellbeing. However, participation in class activities such as singing, dancing, music, exercise, or other activities is statistically significant for all three measures of wellbeing, suggesting that engagement in light learning activities is associated with better wellbeing. These results can serve as starting points for policy discussions as they highlight the potential of lifelong learning for improving the quality of life of older adults.

This study makes three contributions to the existing literature. First, this paper introduces a novel index to comprehensively measure subjective wellbeing of the elderly population, and thus, contributes to the advancement of measurement tools in this domain. Second, this paper provides new empirical insights, based on a primary survey we conduct in Singapore, that adds to the global literature on the roles of lifelong learning in improving subjective wellbeing of elderly people. Third, the study contributes by demonstrating the use of use of machine learning for the study of elderly well-being. Besides demonstrating the applicability and value of machine learning tools in the realm of social science research, it also opens avenues for future research to employ similar advanced methodologies in various social research topics.

It is important to emphasize that our objective is to explore whether an association between lifelong learning attitudes and elderly well-being exists, and not attempt to identify the causal effect that lifelong learning attitudes may have on well-being. Therefore, our results should be interpreted from a correlation, not causal perspective.

The rest of the paper is as follows. Section 2 explores the literature on lifelong learning behaviors and wellbeing. Section 3 discusses our primary data and the approach of constructing an index (called the Multidimensional Welfare Index) as a measure of subjective wellbeing. Section 4 presents the exploratory analysis of our data. Sections 5, 6 and 7 discuss the results from the MCA, decision tree, and regression analyses, respectively. Section 8 compares the findings with existing research. Section 9 concludes and discusses future research directions.

## 2. Background

This section explores the relationship between lifelong learning behaviors and wellbeing of older adults. We will begin by identifying and discussing the key factors that determine the subjective wellbeing of older adults. Next, we will examine the concept of lifelong learning behavior, its definition, and the effects it has on older adults. Finally, we will delve into existing research on the relationship between lifelong learning behavior and its impact on the elderly population in Singapore.

### 2.1 Determinants of subjective wellbeing

As the population ages, it is important for policymakers to address the risks to wellbeing among the elderly. This is a difficult issue as many factors, both innate and external, can affect one’s wellbeing. A summary of the literature examining determinants of subjective wellbeing is provided in Table 1.

**Table 1:** A summary of literature examining determinants of subjective wellbeing (in chronological order).

Author (Year)	Sample	Methodology	Findings on determinants of wellbeing
[9] Costa and McCrae (1980)	1100 men surveyed via mails in 1976	Correlation analysis	Happiness is positively correlated with extraversion, and negatively correlated with neuroticism
[10] Pinquart and Sörensen (2000)	286 empirical studies on the association of socioeconomic status, social network, and competence with subjective wellbeing in the elderly	Meta analysis	Income is correlated more strongly with subjective wellbeing than is education; quality of social contacts shows stronger associations with subjective wellbeing than the quantity of social contacts.
[11] Diener and Biswas-Diener (2002)	-	Review studies	Income enhances subjective wellbeing; but the effect appears to be little for well-off individuals who have higher material desires.
[12] Evans et al. (2002)	497 aged 60 years or above, living in a rural New York county, were contacted; among which 204 has responded.	Multiple regression	Housing quality is associated with positive affect among the older adults living independently in the community. This relation is mediated by place attachment.
[13] Silverstein et al. (2006)	1,561 parents aged 60 and older living in rural Anhui Province, China were surveyed in 2001	multiple regression	Elderly parents in multi-generational homes, especially with grandchildren or receiving more support from adult children, experienced better psychological well-being due to increased emotional cohesion and remittance benefits.
[14] Friedman et al. (2010)	1,312 Terman participants	Hierarchical linear regression	Neuroticism predicted poorer physical health and subjective wellbeing in old age, with higher mortality risk for women but lower risk for men. Extraversion correlated with improved social competence in both genders.
[15] Ní Mhaoláin et al (2012)	466 older people living in a community in Dublin	Stepwise regression model	Depression, loneliness, neuroticism, extraversion, physical activity, age and self-reported exhaustion were predictors of life satisfaction.
[16] Teerawichitchainan et al., 2015).	3042 people from 2012 Myanmar Aging Survey; 2310 people from 2011 Vietnam Aging Survey; 16015 people from 2011 Survey of Older Persons in Thailand	sequential, incremental ordinary least square (OLS) regressions	Living with a culturally preferred gender child boosts emotional health for Vietnamese and Thai elders. In Myanmar, psychological well-being differences across living arrangements are minimal, except for coresidence versus network arrangements.
[17] Al-Butmeh and Al-Khataib (2018)	a convenience sample of 300 people aged 65 years and older living in the Bethlehem district	stepwise regression analysis	Chronic diseases have negative effect on psychological health and quality of life
[18] Etxeberria et al., (2019)	102 community-dwelling healthy older adults aged between 85 and 104	Correlation and stepwise hierarchical linear regression	Neuroticism and extraversion are the strongest predictors of life satisfaction. Subjective wellbeing was mainly predicted by personality traits.

#### 2.1.1 Personality–neuroticism vs. extraversion

There are studies that consider whether subjective wellbeing is related to personality types. [9] examined the relationship between personality and subjective wellbeing in their study, and found that happiness is positively correlated with extraversion personality and negatively correlated with neuroticism. A possible explanation is that individuals with extraverted personality tend to be more sociable, actively involved in social activities, and have greater participation in their community. These traits, in turn, are associated with increased enjoyment of life, which leads to a higher level of happiness and satisfaction with life. Conversely, individuals with neurotic personalities tend to display higher levels of anxiety, guilt-proneness, and psychosomatic disorders. These traits may lead to a greater susceptibility to negative feelings and experiences, which can negatively impact overall life satisfaction [9].

The claim that extraverted personalities are positively correlated with wellbeing while the converse is true for neurotic personalities is supported by multiple studies, such as [14, 15]. These studies found that people with neurotic personalities tend to focus more on the negative aspects of their lives, making them more vulnerable to various forms of psychopathology, such as stress, anxiety, depression, and emotional instability [18, 19]. These traits can have a negative impact on work performance and relationships with family and friends [19]. As a result, people with neurotic personalities are more likely to have lower evaluations of their life satisfaction and have difficulties integrating into society.

#### 2.1.2 Socioeconomic status (SES)

Questions have also been raised as to socioeconomic status matter for one’s wellbeing. A meta-analysis which examined the association of socio-economic status (SES), social network, and competence with the subjective wellbeing of older adults, concluded that income has a positive influence on subjective wellbeing [10]. Specifically, it was found that individuals with higher income levels have access to more opportunities and a larger variety of commercial goods, which can contribute to a higher quality of life. By contrast, low-income earners may face financial hardships and economic stress, which can negatively impact their mental health and subjective wellbeing. This highlights the importance of income level as a determinant of the mental health and wellbeing of older adults.

Another study [11] also supports the claim that income affects subjective wellbeing. The study found that individuals with higher income levels are able to move out of poverty and meet their basic needs such as food, shelter, and security [20]. Moreover, the attainment of a higher standard of living and reduced stress due to financial difficulties can lead to better mental health and higher levels of subjective wellbeing.

While an increase in income may lead to improve wellbeing, its impact could be heterogeneous among different income groups. For instance, [11] also observed that an increase in income has a greater impact on subjective wellbeing for low-income earners than for middle to high-income earners. For individuals who are already well-off, an increase in income has a limited impact on improving their subjective wellbeing as their basic material needs have already been met. In some cases, an increase in income for these individuals may even have a negative impact on their subjective wellbeing, as they may experience decreased enjoyment of small pleasures and increased stress in accumulating wealth [21, 22]. These studies show that the mechanisms that affect wellbeing can be highly complex.

#### 2.1.3 Illness and environment

The state of mental and emotional health in seniors can be influenced by factors ranging from chronic diseases, lack of intergenerational support, living arrangements, to the built environment. According to [17], chronic diseases can lead to mental health issues such as major depression, post-traumatic stress disorder, and obsessive-compulsive disorder. Another study by [13] found that lack of intergenerational support, such as exchanges of financial, instrumental, and emotional support between parents and their adult children may adversely affect the mental health and wellbeing of older persons. Living with family members can protect against social isolation, although in certain cultural context, there could be increased risk of tension among family members arising from conflicts between wives and mothers-in-law for example [16]. The built environment is also important for promoting the independence of seniors and stronger social attachments, such as feelings of security and belonging, as highlighted in [12].

### 2.2 Adult learning and wellbeing

Researchers have explored the link between adult learning and wellbeing and health, primarily in western contexts. Common findings indicate that continuous learning positively affects the physical and psychological health of older adults (for example, [23] for the US, and Hammond [24] for the UK). A summary of the literature examining relationship between adult learning and subjective wellbeing is presented in Table 2.

**Table 2:** A summary of literature examining relationship between adult learning and subjective wellbeing (in chronological order).

Autor (Year)	Sample	Methodology	Findings on the relationship between adult learning and wellbeing
[23] Panayotoff (1993)	114 subjects registered in four programs offered for older adults at a community college	measures‐design analyses of variance	Only some continuing education programs have positive short‐term effects on the health of older adults.
[25] Dench and Regan (2000)	336 people aged 50 to 71	Structured face-to-face interviews	Benefits reported by learners include self-confidence, self-esteem and satisfaction with life.
[24] Hammond (2004)	145 adults	In-depth biographical interviews; group interviews	Educational experiences had positive effects upon health outcomes only when they matched the interests, strengths and needs of the learner.
[26] Narushima (2008)	Case study of one traditional program for seniors run by a school board in Ontario	Interviews, classroom observations; documents	Affordable and accessible public continuing education program played a vital role for retirees, especially seniors at risk.
[27] Leung and Liu (2011)	cross-section data of 1003 older adults in China	Multiple regression analysis	Self-efficacy and continuing education are associated with good quality of life in learners aged 60 and above.
[28] Escuder-Mollon (2012)	72 senior learners at Senior Citizens’ University (SCU) in Spain in 2010/2011 academic year	Open interviews	Perceived quality of life is strongly related to one’s ability to integrate into an ever-changing society.
[29] Thang et al (2012)	64 older adults aged 50 to 64 years old in Singapore	in-depth semi-structured interviews	Lifelong learning impacted older adults’ well-being in various ways (intellectual, social, physical, emotional, spiritual, and occupation wellness).
[3] Jenkins and Mostafa (2015)	3096 observations from four waves of data from the English Longitudinal Study of Ageing	Fixed effects panel regressions	Informal learning was associated with higher levels of psychological wellbeing. Formal learning was not found to be associated with higher wellbeing.
[30] Goh et al. (2019)	4549 Singaporeans aged 60 years and above in 2016–2017	mixed-methods evaluation	Perceived benefits of learning include enhanced personal development, gain in social capital and fulfillment of learning outcomes.
[31] Suyono (2021)	Indonesian Silver Colleges	Participant observation and in-depth interviews	Encouraging lifelong learning among the elderly can promote their inclusion in communities, recognise their contributions, reduce risks of isolation and challenge age discrimination.

Dench et al. [25] conducted a study where they interviewed 336 older adults over a two-year period and found that about 70% of them had completed some form of learning, and of those, 80% reported that learning had improved their life satisfaction, self-confidence, self-perception, and ability to cope. Narushima [26] conducted a small-scale interview study and found that educational programs specifically designed for people aged 60 and above in Canada were beneficial for health (mainly psychological) through enduring interest, informal social support networks built in the classroom, and raised awareness of the right to learn.

While these studies have provided some insights into the benefits of learning among seniors, they are mostly qualitative in nature. Quantitative studies using larger scale datasets have been conducted, but such studies are limited. [27] studied a cross-section dataset of 1003 older adults in China and found that lifelong learning may improve their quality of life, particularly in terms of self-efficacy. They also found that it was not the number of courses taken by the older adults that mattered, but the continuation of learning that was important. Additionally, [3] used four waves of data from the English Longitudinal Study of Ageing to examine the relationship between participation in learning and the psychological wellbeing of older adults in England and found that informal learning was associated with higher levels of psychological wellbeing.

Why would lifelong learning benefit wellbeing? In a study on the impact of lifelong learning on quality of life, [28] explained that the perceived quality of life is strongly related to one’s ability to integrate into an ever-changing society: people who are unable to keep up with societal changes, such as technological advancements, political and economic changes, may face exclusion within their community. Therefore, by acquiring lifelong learning behaviors, [28] argued individuals are able to stay updated and relevant, keeping them integrated into society for longer periods of time.

Similarly, [31] explained that the quality of life of the aged population is strongly related to societal inclusion. He showed that lifelong learning scheme under the community organisations, university social services learning programme and Silver Colleges had improved the elderly’s wellbeing and encouraged them to lead an active life. Therefore, lifelong learning leads to better wellbeing in that it promotes the inclusion of older adults in communities, recognises their contributions, reduces the risks of their isolation and challenges age discrimination.

### 2.3 Studies in Singapore context

Singapore is facing a rapidly aging population. By 2030, one in four Singaporeans will be aged 65 years and above [32]. This raises concerns about the need to help older adults age successfully in the face of a rapidly changing world [4].

Studies on the relationship between lifelong learning and elderly wellbeing in the context of Singapore are limited. [29] conducted a study on older adults’ experiences and perceptions of lifelong learning, and the impacts of lifelong learning among older adults in Singapore. They found that learners have a positive perspective of lifelong learning, who viewed continuous learning as beneficial not only to increasing their own wellbeing, but also in contributing back to society. Interestingly, the study also found that many non-learners (41% of the non-learner respondents), i.e., those who did not attend lessons, are aware of the importance of learning and interested in learning [33]. This suggests that there are barriers preventing older adults from participating in lifelong learning opportunities.

This study contributes to this strand of literature by adding empirical evidence from the Singapore perspective. By collecting primary data from 300 individuals aged 65 and above in Singapore, we aim to add context-specific evidence and provide deeper understanding of how lifelong learning is associated with the various types of wellbeing of the elderly. Findings of our study will offer valuable insights for both local and global policymakers.

## 3. Data and methods

All procedures performed in this study were in accordance with the ethical standards of the Singapore University of Social Sciences (ref: APL-0148-2022-EXP-01) at which the studies were conducted.

### 3.1 Primary survey

Our study contributes to the literature by examining the association between lifelong learning attitudes and subjective wellbeing based on a primary survey of 300 older adults aged 65 and above in Singapore. We adopt a convenience sampling approach to survey across diverse locations across Singapore so that our data encompasses seniors from a spectrum of neighborhods. Our power analysis, which is based on a significance level of 5% at the current resident size of those aged 65 and above (678,133 in 2022), suggests that a sample size of 300 observations will be adequate for the proposed study. The survey aims to capture information about older adults’ lifelong learning behavior and their subjective assessment of their mental and emotional state. The survey questions are categorized into three groups.

The first part of the survey collects social and demographic data. Respondents were asked about their marital status, number of children, educational level, income level, whether they were working and the types of work they were engaged in (if any), whether they have any physical impediments such as mobility problems and disability, and the quality of their relationships with family and friends.

The second part of the survey collects information on the older adults’ attitudes towards lifelong learning such as their willingness to learn (purpose of learning, or reasons for not learning), learning patterns (participation, areas of study, and frequency of learning), concerns in taking on lifelong learning (fees, course designs, and feasibility), and perceived impact of participation in learning. Respondents were also asked if they have participated in class activities such as singing, dancing, music, etc. or visited places such as a museum, library, educational institute, etc. Additionally, the survey asks about their attitudes towards lifelong learning, whether lifelong learning is important to them, why it is important (or otherwise), and the barriers they encounter in learning.

The third part of the survey collects information on various measures of subjective wellbeing, namely, “Quality of Life", "Satisfaction with Life", and "Psychological Wellbeing" so as to capture a comprehensive understanding of the older adults’ subjective wellbeing. Quality of life provides a comprehensive view of various life domains, including physical health, mental health, social relationships, financial well-being and environmental factors; satisfaction with life focuses on an individual’s overall assessment of their life as a whole; and psychological well-being emphasizes positive mental health and psychological functioning. While there is overlap among these concepts, each measure provides a distinct perspective on the holistic experience and mental state of an individual. This study uses these concepts in combination to gain a comprehensive understanding of an individual’s overall well-being.The “Quality of Life” measure is evaluated using the CASP-19 construct, which contains four domains, namely, Control, Autonomy, Self-realisation and Pleasure, with a total of 19 questions. It is specifically designed to assess the quality of life of individuals in early old age and widely used in the aging surveys such as the English Longitudinal Survey of Ageing [34]. Respondents were asked to rate on a scale of "Often", "Sometimes", "Not often", and "Never" for 19 statements related to "*how frequently in the past week you have experienced the following* [statements]." For questions related to "Satisfaction with Life", respondents were given five statements related to their satisfaction about their current living conditions, their future outlook, and past regrets, which they were asked to rate on a scale of 1 (strongly disagree) to 7 (strongly agree). It is often used in the litearture ot measure global cognitive judgements of one’s life satisfaction [35, 36]. For the "Psychological Wellbeing" measure, we use the 5-item World Health Organization Well-Being Index (WHO-5), where respondents were asked to rate five statements related to "*how you have been feeling over the past two weeks*" on a scale of 1 (All of the time) to 6 (At no time). It was first published in 1998 and widely used in the literature to assess subjective psychological wellbeing [37]. The survey questions related to “Quality of Life”, “Satisfaction with Life” and “Psychological Wellbeing” are provided in Table A1 in S1 Appendix. This study has obtained approval from Singapore University of Social Sciences Instituional Review Board (SUSS-IRB) with the reference number APL-0148-2022-EXP-01. The survey was conducted during 15^th^ Jun 2022 and 15^th^ Sep 2022, and respondents have given written informed consent to participate in the survey.

### 3.2 Measuring wellbeing

The wellbeing of a person could be determined by many factors. To summarize the responses, we follow the approach of Sarma (2008) to construct an index, which we call the **Multidimensional Wellbeing Index** (MWI), for each set of wellbeing measure (Quality of Life", "Satisfaction with Life", and "Psychological Wellbeing"). Unlike using the total scores of the responses for each wellbeing category, the MWI has certain properties such as being unit free, bounded, monotone, and scale invariant, that simple measures like the summation (or average) does not have.

To construct such an index, which aggregates many different measures into an overall index with the said properties, the idea is to first construct an index at the disaggregated level. Specifically, for each measure to be included in the overall index, we first convert it into an index with the said properties before aggregating all the indices into an overall index. This approach effectively preprocesses the measure into an index first before they are aggregated.

To elaborate let *h* indicate if MWI measures "Quality of Life", "Satisfaction with Life", and "Psychological Wellbeing", *k*_*h*_ be the number of questions related to subjective wellbeing measure *h*, and *i* be the index of the survey respondent. The MWI is constructed as

$$MWI_{h}^{i}=\frac{1}{2}\left(\sqrt{\frac{\sum_{j=1}^{k}d_{ijh}^{2}}{k_{h}}}+(1-\sqrt{\frac{\sum_{j=1}^{k}\left(1-d_{ijh}^{2}\right)}{k_{h}}})\right)$$ [1]

where *d*_*ij*_ measures person *i*’s subjective wellbeing (*h*) based on the *j*th dimension, and is given by

$$d_{ij,h}=\frac{A_{ij,h}-m_{j,h}}{M_{j,h}-m_{j,h}}$$ [2]

where *m*_*j*_ and *M*_*j*_ are the lower and upper bound of the survey response on the *j*th question related to wellbeing (*h*). Compared to the simple summation of the ordered responses that is widely used in the literature, the proposed MWI has the following desirable properties associated with index number analysis, i.e., it is

1. unit free, so that we may compare across different individuals.
2. bounded between 0 and 1 (where 0 is the worst wellbeing outcome and 1 is the best), so that the index is easy to interpret.
3. monotone, so that the whole index will increasing if wellbeing is reported to be better in one or more dimensions.
4. homogeneous, so that if each dimension in the MWI is changed by a constant amount, it should not change the value of the index. Such a property is also known as scale invariance and is important as the scale of the responses should not affect the MWI.

The MWI is easy to interpret as it summarizes the relevant factors related to subjective wellbeing into a single score. Individuals with higher MWIs have higher levels of subjective wellbeing.

Our paper is not the first to employ a multidimensional index approach. A notable example is the work of [38] in the financial inclusion literature, where a similar methodology to ours, based on [39], was employed to construct a financial inclusion index. Additionally, unlike Principal Component Analysis, the multidimensional index we adopt does not aim for dimension reduction; instead, it attempts to encompass all relevant dimensions associated with the wellbeing concept it captures.

While the implementation of index number approaches may not be commonplace in the wellbeing literature, its successful utilization in other domains, together with the favorable properties of index numbers, motivated us to adopt this approach for measuring wellbeing in our study. We believe this methodological choice aligns with the goal of summarizing diverse wellbeing responses while preserving key properties crucial for accurate representation.

Furthermore, adopting the multidimensional index approach serves to mitigate potential biases inherent in the conventional mean response method, which appears to overly emphasize positive wellbeing outcomes. Nevertheless, our findings remain robust regardless of whether the Multidimensional Wellbeing Index (MWI) or a simple calculation of response scores is employed. The detailed analysis using the conventional index approach is available from authors upon request.

For future research, it is important to emphasize that the multidimensional welfare index approach is not an approach that is only specific for measuring wellbeing. It is called a “welfare” index because welfare is the focus here. Research that seeks to construct an overall outcome measure based on several other measures may consider the multidimensional index used here as a possible approach for doing so.

### 3.3 Measuring lifelong learning

We use three measures to measure lifelong learning. Our main variable, *learn_elderly*, contains the response “yes” or “no” by the elderly to the question: “Should the Elderly Keep Learning?”. We refer to this response as indicating the elderly’s attitude towards lifelong learning.

We employ two further indicators of wellbeing related to activities that the elderly had undertaken. The first measure is *class_total*, which measures the total number of different classes taken in the last 12 months. In the primary survey, we asked the elderly if they had taken classes in singing, dancing, music, exercise and others in the last 12 months. We then sum up the total number of different classes the elderly had taken as *class_total*.(e.g. if they had taken singing and dancing classes, then *class_total* will have a value of 2). The second measure is *places_total*, which measures the total number of different places the elderly has been to in the last 12 months. The places include gym, sports club, museum, library, educational institute, and others. The idea is that these indicators measure some aspects of light engagement in learning, for example, learning to dance, a new song, visiting the library, etc., and thus, they provide additional information about learning beyond learning attitudes. The full list of variables employed in this study can be found in S1 Dataset.

## 4. Exploratory data analysis

We first explore our data by using both univariate and bivariate analysis and present selected data visualisations in this paper. We then employ unsupervised and supervised machine learning techniques to study the association between lifelong learning and the mental and emotional wellbeing of the elderly. To do so, we consider two general approaches–multiple correspondence analysis (MCA) (i.e., unsupervised) and decision trees and regression (supervised). The MCA will be used to reveal the association between the variables in the dataset, as it is especially valuable in dealing with categorical data, reducing the dimensionality of the data, exploring patterns in the data, and visualizing relationships. The decision tree and regression will be used primarily to uncover which factors are important for lifelong learning. The decision tree analysis enables automatic selection of the most relevant factors for prediction; it allows non-linear relationships between predictors and outcomes, and its visual representation makes the interpretation of results easy and straightforward. Regression analysis complement the decision trees by providing a more refined estimate on the significance and effect size of the predictors. Combining the unsupervised machine learning method (MCA) and the supervised machine learning techniques (decision tree and regression) enables a comprehensive and nuanced understanding of the data. To save space, we present the key data visualizations. A comprehensive set of analyses is available upon request.

### 4.1 Univariate analysis

**Fig 1:**
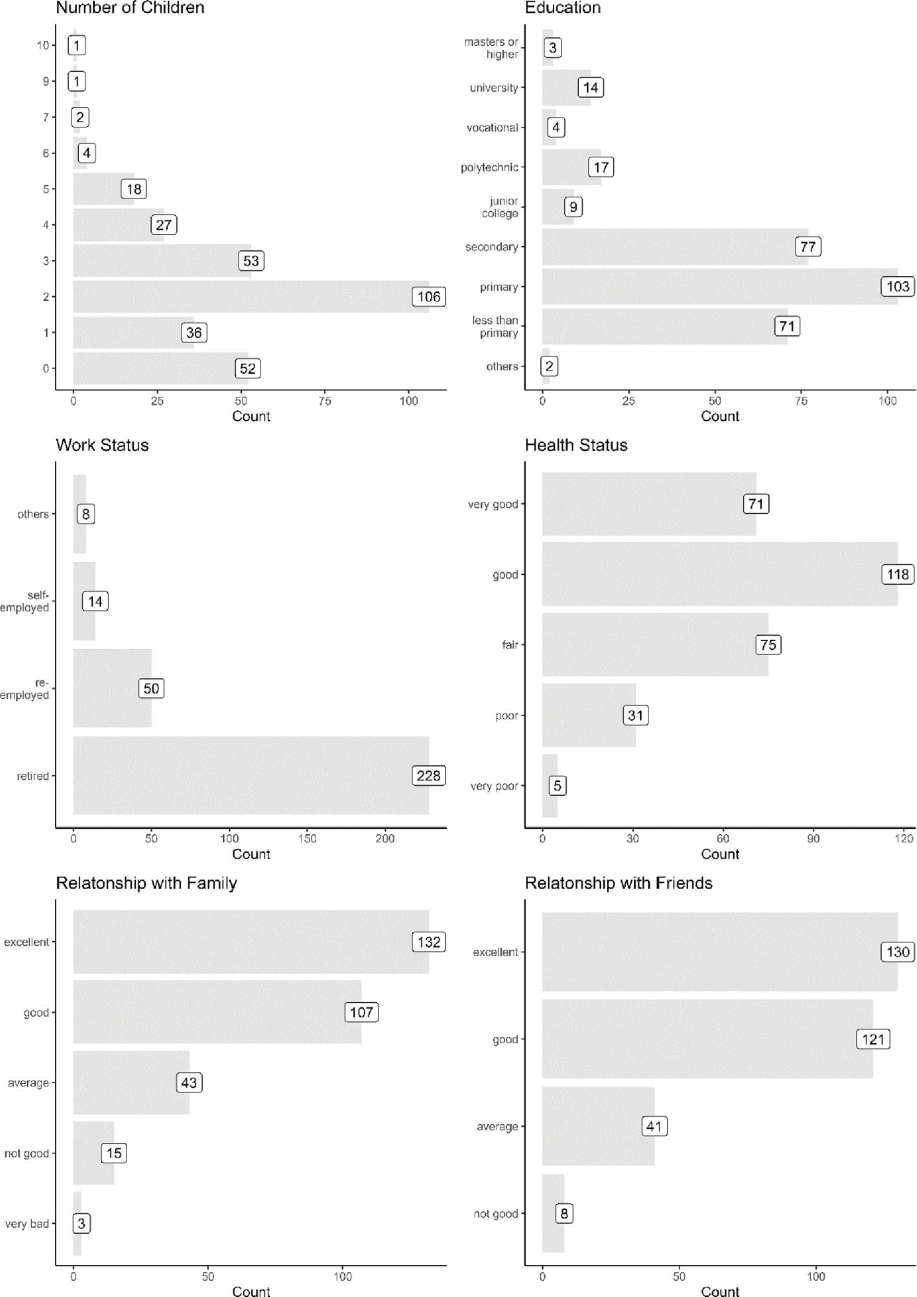
Profile of the survey respondents.

We first begin by summarizing the demographic attributes of our survey respondents in Fig 1. As the figure shows, most of the survey respondents have 2 children (106 respondents) and primary education (103 respondents). Most of them are also retired (228 respondents) and reported to be in relatively good health (189 respondents reported their health as “good” and “very good”). Additionally, most reported having good relationships with their family and friends.

**Fig 2:**
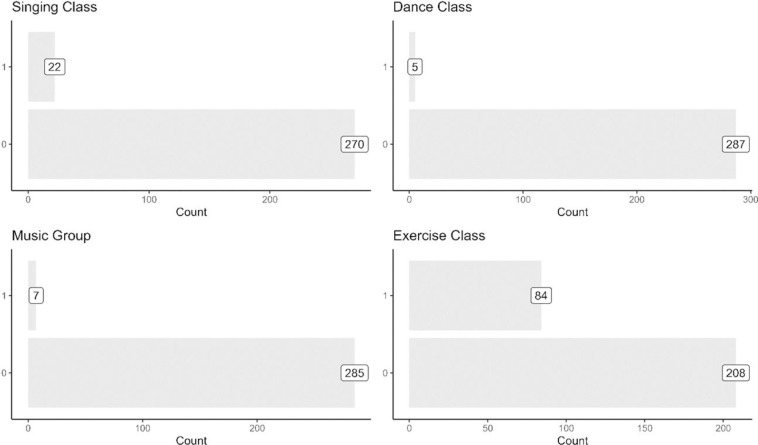
Classes participated in the last 12 months.

**Fig 3:**
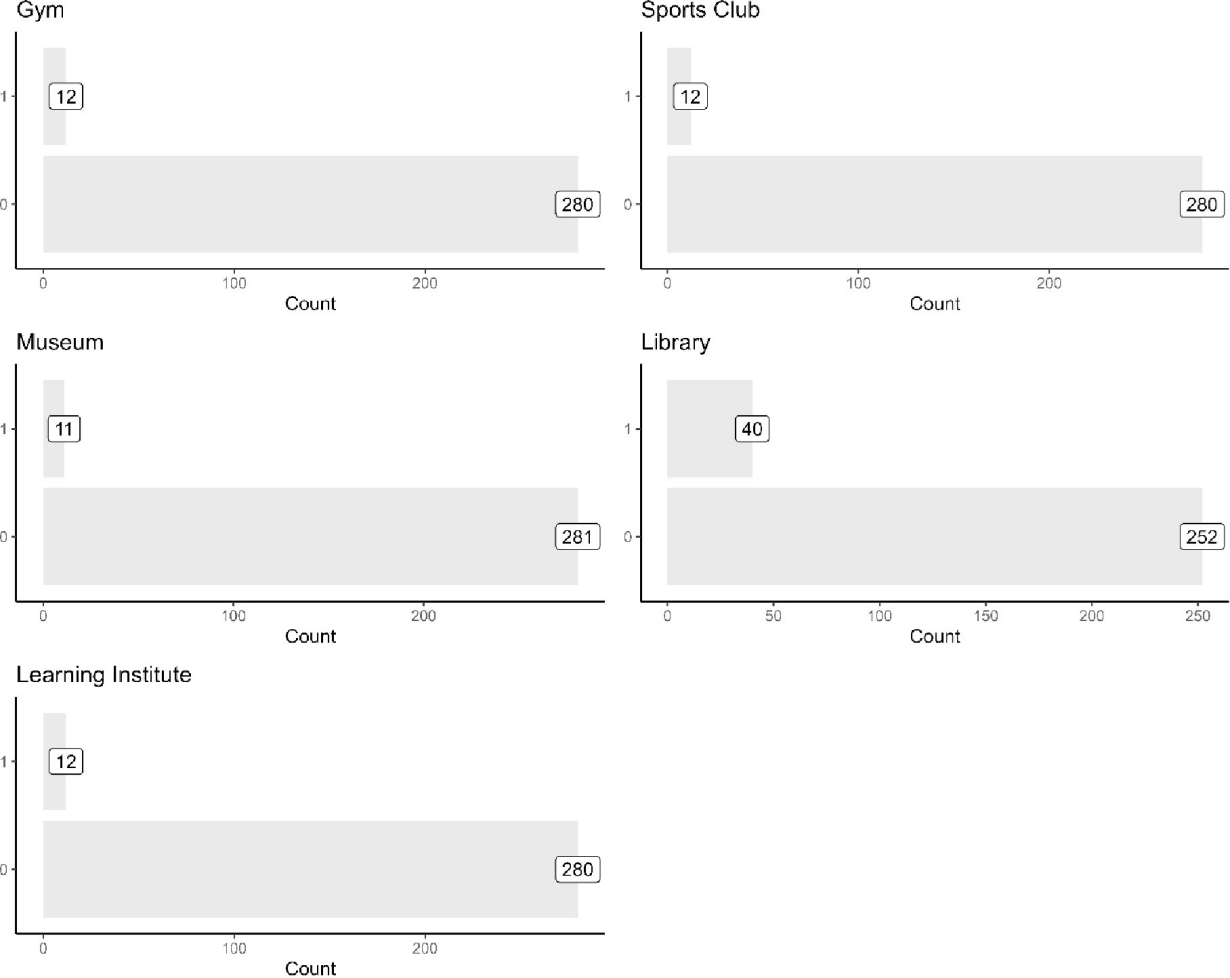
Visited an exercise, cultural, and learning place in the last 12 months.

To explore how active the respondents are, Fig 2 shows the types of classes the respondents had participated in the last 12 months. The most popular class activity is exercise (84 respondents), followed by singing. Only a small number of respondents had participated in a dance or music class. Among the respondents, 147 of them did not participate in any activity in the last 12 months, 118 participated in exactly one activity, and 27 participated in two or more activities. Compared to class activities, Fig 3 shows that fewer respondents reported to have visited a place for exercise (gym or sporting club), culture (museum) and learning (library or education institute) in the last 12 months. During this period, 225 respondents did not visit any of the abovementioned places, while only 52 respondents visited only one place, and 15 visited two or more places. The library is the most popular place visited by the elderly (40 respondents), while a much smaller number had visited a gym, sports club, museum, or an educational institute.

The observation that more elderly respondents were involved in class activities than visiting places could be due to challenges in mobility and personal independence. Among those with mobility difficulties (not shown in the figures below), 17 respondents had participated in a class activity in the last 12 months, but only 6 respondents had visited a place (for exercise, cultural or learning), suggesting that it is more challenging for persons with mobility issues to visit places than to participate in classes.

**Fig 4:**
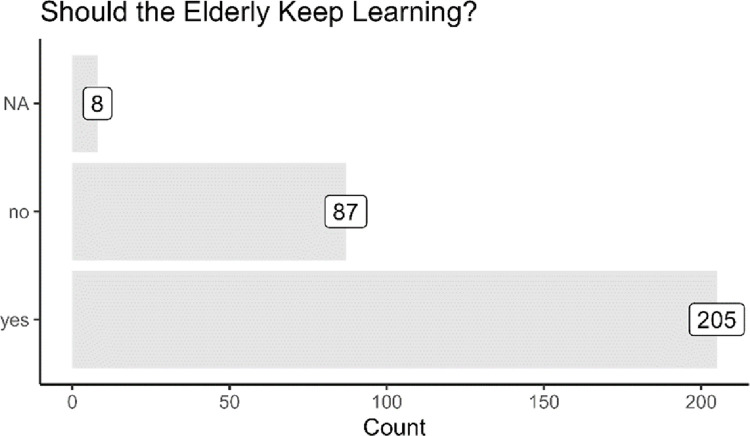
Attitudes towards lifelong learning.

Finally, we present the elderly’s attitudes towards lifelong learning. As Fig 4 shows, 87 respondents felt that “Elderly people do not need to learn” as opposed to 205 who felt that “Elderly people should keep learning” (8 respondents did not answer this question). Therefore, most respondents in our sample felt that lifelong learning was important.

As alluded previously, attitudes towards lifelong learning could be related to mobility and disabilities. We found that those who have mobility issues or disabilities tend to feel more negatively towards learning. For instance, without considering mobility challenges, about 30 percent of respondents felt that “Elderly people do not need to learn” (i.e., 88 out of 301). However, conditioning on those with mobility difficulties, the percentage of respondents who felt negatively towards learning rises to 40.7 percent. This suggests that attitudes towards lifelong learning could be associated with the physical state of the elderly.

### 4.2 Bivariate analysis

**Fig 5:**
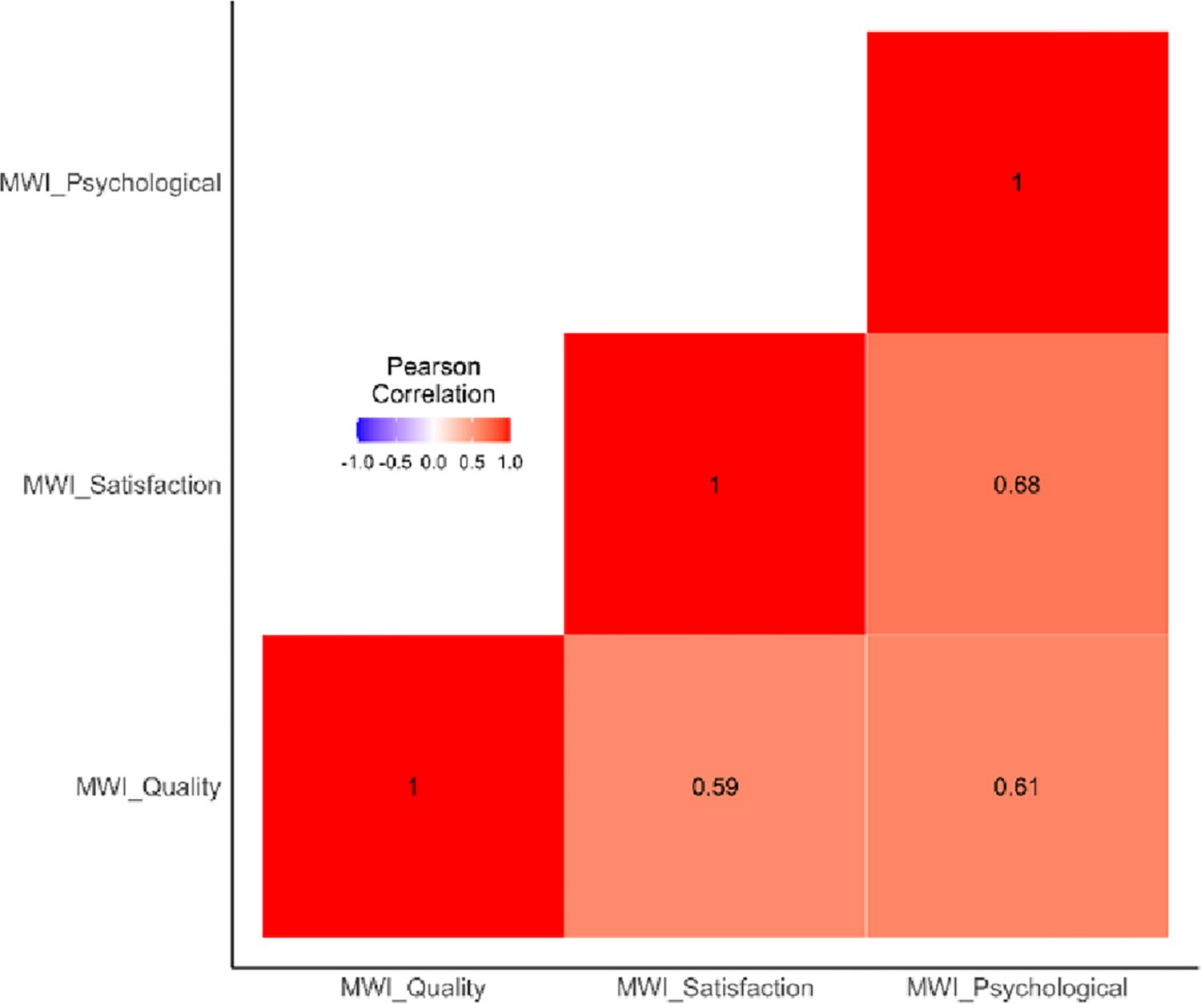
Cross-correlations of the MWI measures.

Fig 5 presents the correlation between the three multidimensional welfare index (MWI) measures–“Quality of Life” (MWI_Quality), “Satisfaction with Life” (MWI_Satisfaction), and “Psychological Wellbeing” (MWI_Psychological). Each index ranges from 0 to 1, where 1 indicates the greatest level of subjective wellbeing.

As Fig 5 shows, the cross-correlations of the three MWI measures range from 0.59 (Quality and Satisfaction) to 0.68 (Satisfaction and Psychological), suggesting a medium level of correlation. While it shows the relevance of the three indices in measuring the well-being, the MWI measures are themselves not overly strongly correlated. This suggests that these 3 measures capture a certain unique aspect about wellbeing.

**Fig 6:**
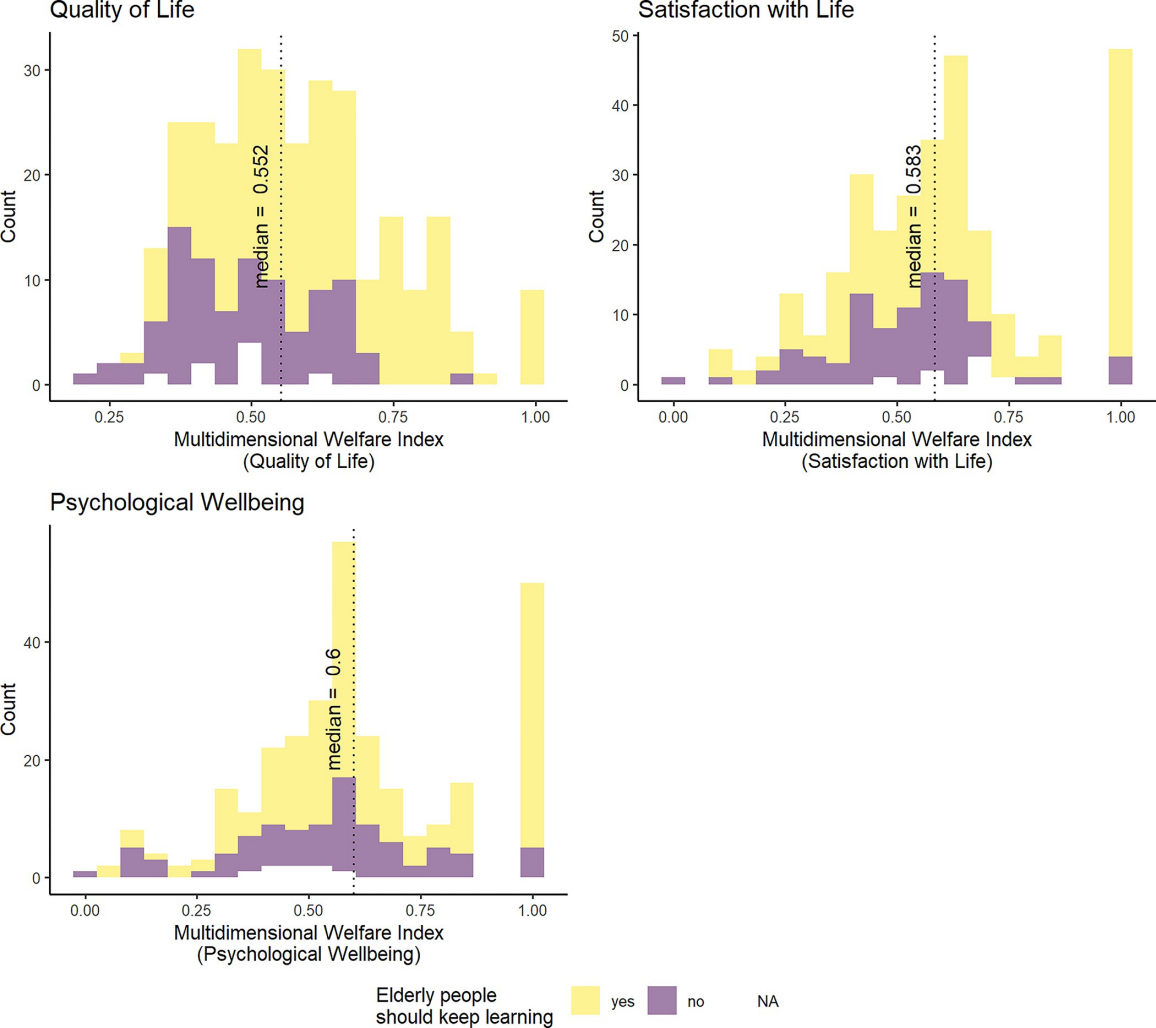
Wellbeing and lifelong learning.

Does the distribution of subjective wellbeing of the elderly depend on attitudes towards lifelong learning? In Fig 6, those who reported that the elderly do not need to learn tend to be skewed more towards the left, i.e., towards lower levels of wellbeing. The skewness is more apparent for “Quality of Life” and “Satisfaction with Life”, although not for “Psychological Wellbeing”. This suggests that positive attitudes towards lifelong tend to be associated with the better levels of reported wellbeing, although this may not be true for psychological health.

## 5. Multiple correspondence analysis

We implement Multiple Correspondence Analysis (MCA) to explore, if any, the relationships between lifelong learning, wellbeing, and other demographic attributes. MCA is a dimensionality reduction technique specifically designed for categorical variables. It aims to condense the information from a large number of categorical variables into a smaller set of variables called factors. This process is similar to Principal Component Analysis (PCA), which is commonly used for continuous data. However, MCA distinguishes itself by being tailored for categorical data. Since our survey responses are mostly categorical data, it would be appropriate for us to employ MCA to study how the responses are associated and especially how the attributes of the elderly are associated with their attitudes towards lifelong learning.

To represent categorical variables, MCA utilizes a technique called one-hot encoding. In this context, each category of a variable is transformed into a binary vector using this encoding, where each category is represented by a binary (0 or 1) indicator variable. The dataset is then structured as a cases-by-variables indicator matrix.

Unlike PCA, which employs eigenvalue decomposition, MCA utilizes singular value decomposition (SVD) to achieve orthogonality and dimensionality reduction. The procedure begins by transforming associations between categories of discrete variables into coordinates within a multidimensional space, employing the previously mentioned one-hot encoding. After one-hot encoding, scores are assigned to each category of discrete variables, where the summation of these scores is then maximized under certain constraints.

**Fig 7:**
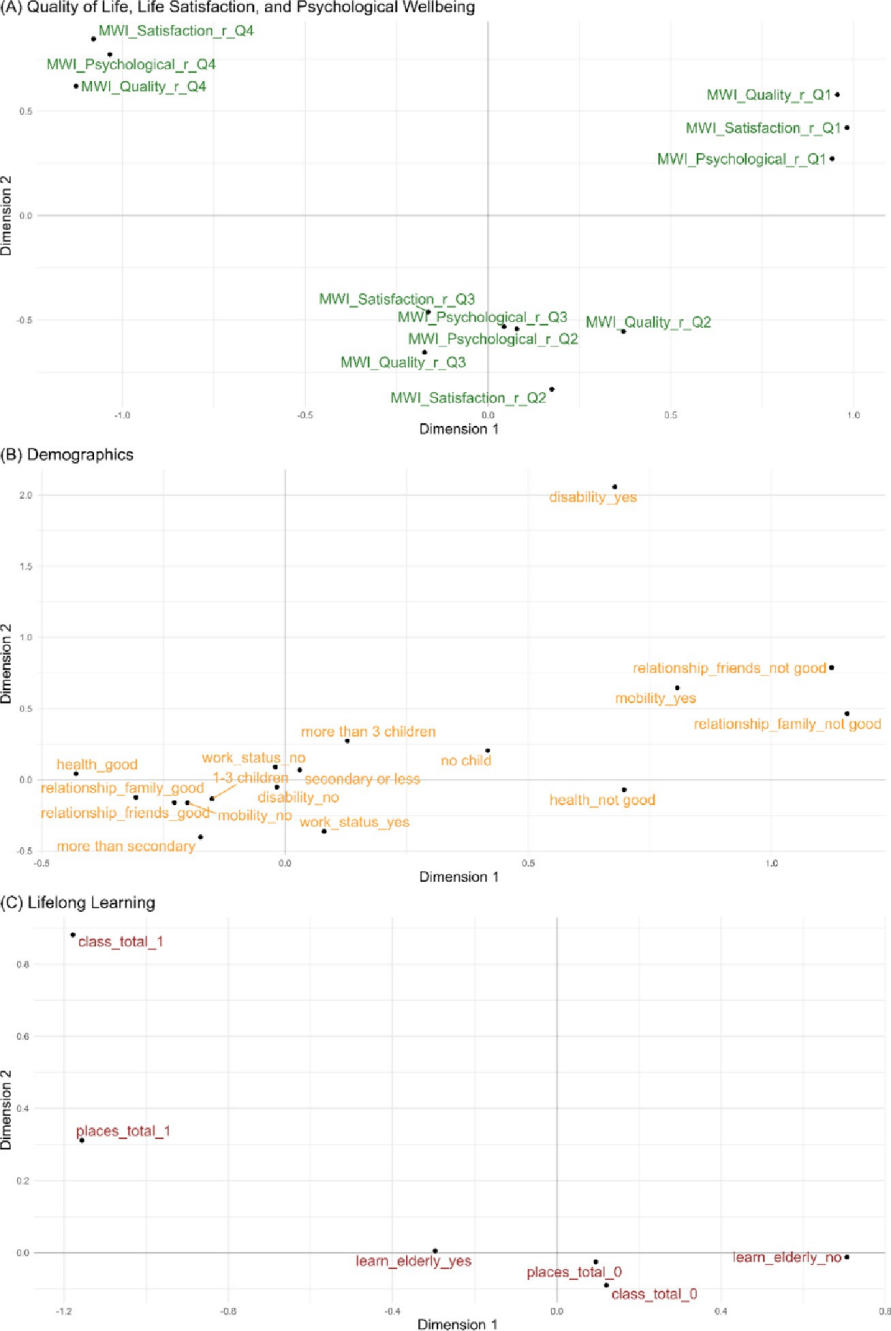
Factor maps on dimensions 1 and 2.

**Fig 8:**
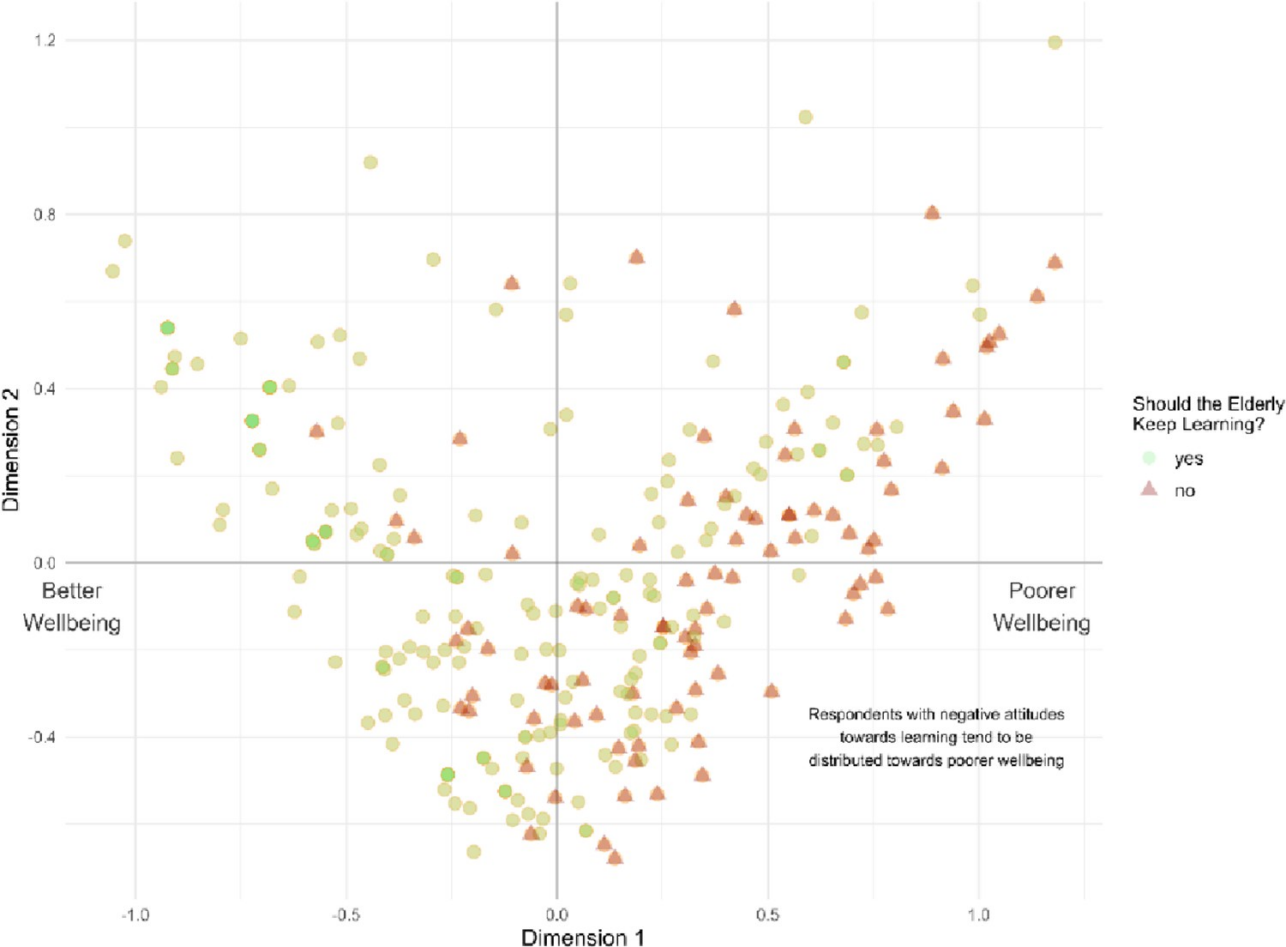
Attitudes towards lifelong learning and wellbeing.

The resulting dimensions, or factors, in MCA capture both the associations between variables and the proximity between individuals. Visualization of the data is often performed to interpret the results (see Figs 7 and 8 below). In these visualizations, points in the same direction from the origin reflect high associations, points around the origin represent the mean, while points away from the origin indicate deviations from the mean. These visual cues assist in understanding the relationships and patterns within the categorical data. For a full treatment of MCA, please refer to Chapter 18 of [40].

### 5.1 Data preparation

Before we perform an MCA, we must convert all inputs into categorical variables as MCA can only be applied to categorical data (whose categories are one-hot encoded to implement MCA).

First, we discretize the MWI measures. To do so, we divide each MWI measure into four quartiles. The first quartile, which is tagged by “_r_Q1” (i.e., ranked first quartile), reflects the worst levels of subjective wellbeing. Conversely, the fourth quartile, which is tagged by “_r_Q4” (i.e., ranked fourth quartile), reflects the best.

As a numeric variable, the number of children must also be discretized to conform it to MCA. Thus, we recode it into class values of "no child", "1–3 children", and "more than 3 children". We also recode the total number of different classes taken and the total number of different places the respondents had been to in the last 12 months. For the total number of classes or places been to, we binarize each of variable by assigning a value of 1 if the respondent had taken two or more class or had visited two or more exercise, cultural and learning places. This is to identify individuals who had demonstrated a greater interest in learning (e.g., a person who is keener to learn is likely to try out different classes or visit different places).

We also simplify several categorical variables by combining categories that are similar. For work status, we recode its categories into class values of “yes” for those who are still working and “no” for those who are not. Here, “working” refers to those who are working, working part-time, at an ad-hoc basis or freelance basis. For health status, we recode its categories into class values of “good” (for those who reported “Good” and “Very Good” health) and “not good” (if otherwise). For relationships with family and with friends, we recode its categories into class values of “good” (for those who reported “Good” and “Excellent” relationships) or “not good” (if otherwise). For education, we recode the various levels into “more than secondary” or “secondary or less”. For the generation of the elderly sampled here, education beyond secondary school is considered a high educational achievement, and in our sample, only 43 respondents have more than secondary education while 257 respondents have secondary education or less. The definition of the attributes can be found in Table A2 of the S1 Appendix.

### 5.2 Results

In Fig 7, we present the variable factor map on Dimensions 1 and 2 to visually explore the relationships between variables. These dimensions are the first two principal components, which collectively contribute to 22% of the total variation in our data. A factor map is a graphical representation of the relationships between variables visualized on the principal components as axes. Here, we depict a factor map on Dimension 1 (the horizontal axis) and Dimension 2 (the vertical axis), which are the principal components derived from MCA, where these components reflect the most significant variation in the data. Each variable, whether related to wellbeing, demographics, or lifelong learning, is then represented as a point on the factor map. Categories that are closer to each other in the factor map are more strongly associated. The direction of the points indicates the contribution of variables to the respective dimensions. The position of a category on the map reflects its association with the underlying dimensions, here, Dimension 1 or Dimension 2.

Panel (A) of the factor map, which focuses on the wellbeing variables, reveals some interesting patterns. Specifically, quality of life and life satisfaction exhibit a trend of worsening towards the right-half of Dimension 1 but improve towards the left-half. Intermediate levels of quality of life, life satisfaction, and psychological wellbeing cluster around the center of Dimension 1. This suggests that Dimension 1 can be interpreted as a measure of overall "wellbeing."

Panel (B) demonstrates that poorer relationships with family and friends, along with poorer health, are situated in the right-half of Dimension 1. This implies an association between poor relationships, health, and lower levels of wellbeing. Panel (C) highlights that negative attitudes towards lifelong learning (*learn_elderly_no*) are positioned in the right-half of Dimension 1. Conversely, attributes related to taking multiple classes (*class_total_1*) and visiting different places (*places_total_1*) are located in the left-half of Dimension 1. This suggests that elderly individuals with negative views on learning tend to have poorer wellbeing, while those engaging in classes or exploring various places tend to have better wellbeing.

Although Dimension 2 is more challenging to interpret, it appears to be related to disability. Specifically, the attribute of *disability_yes* is in the upper half of Dimension 2, while *disability_no* is in the bottom half. Additionally, mobility issues (*mobility_yes*) are also located in the upper half. These findings suggest that Dimension 2 may be associated with physical impediments.

In Fig 8, we present the individual factor map that illustrates the factor values of Dimensions 1 and 2 for each respondent in the sample. Scatter points on the plot are differentiated based on individuals’ attitudes towards learning, with positive attitudes represented by light green dots and negative attitudes by dark red triangles.

The figure provides a clear visualization: individuals expressing positive attitudes towards lifelong learning are well-distributed across the entire wellbeing spectrum. Conversely, those with negative attitudes towards learning are predominantly concentrated in the right-half of Dimension 1, which corresponds to lower levels of wellbeing. This underscores the consistent observation that negative attitudes towards learning correlate with diminished wellbeing. Overall, these findings suggest that encouraging positive attitudes towards lifelong learning among the elderly population could be an effective strategy for promoting wellbeing, particularly for those with negative attitudes towards learning.

## 6. Decision trees

To further explore the association between lifelong learning and wellbeing, we construct a decision tree for each MWI measure (i.e., for “Quality of Life”, “Satisfaction with Life”, and “Psychological Wellbeing”).

A decision tree is a non-parametric approach that models how an outcome variable, which is the MWI, is related to a set of attributes or predictors. The model is represented as a tree-like structure, where each internal node represents a decision based on some feature or attribute of the data, and each leaf node represents a predicted outcome.

To construct our decision trees, we utilize the Classification and Regression Tree (CART) algorithm. This algorithm identifies the optimal attribute or predictor to split the data into subsets using binary splits. The goal is to create subsets that are as internally consistent as possible concerning the predicted outcome. The CART algorithm recursively applies this process to each subset, continuing to split the data until the subsets are as internally homogeneous as possible, or, when a predefined stopping criterion is met. At the end of the algorithm, the subsets will primarily consist of respondents who are most similar in their MWI scores as possible, and the attributes leading to a particular subset of individuals, such as individuals with MWI scores, can be read off the decision tree.

Decision trees have gained popularity in recent empirical research as they are easy to interpret. In general, variables higher up in the decision tree are more important for predicting the outcome compared to variables that appear lower down in the tree. This is because the variables at the top of the tree are used to make the initial splits, and thus, have a greater impact on the final prediction than variables that are used in later splits. Therefore, decision trees not only reveal the attributes relevant to the targeted outcome, but also which attributes are more important than others.

However, decision trees are susceptible to overfitting; thus, we must take precautions to prevent the tree from becoming too complex. We accomplish this by “pre-pruning” the tree to restrict its growth. Other pre-pruning approaches include limiting the depth of the tree, imposing a minimum sample size for the leaf nodes, etc. To do so, we restrict each node to have a minimum of 20 observations before considering further splits. If a node has fewer than 20 observations, no further split will be made. As the most important predictors are first used to divide the data, pre-pruning essentially eliminates attributes that are weakly relevant to the outcome variable (i.e. the MWI) at best. The definition of the variables used in constructing the decision trees can be found in Table A3 of the S1 Appendix.

### 6.1 Results

**Fig 9:**
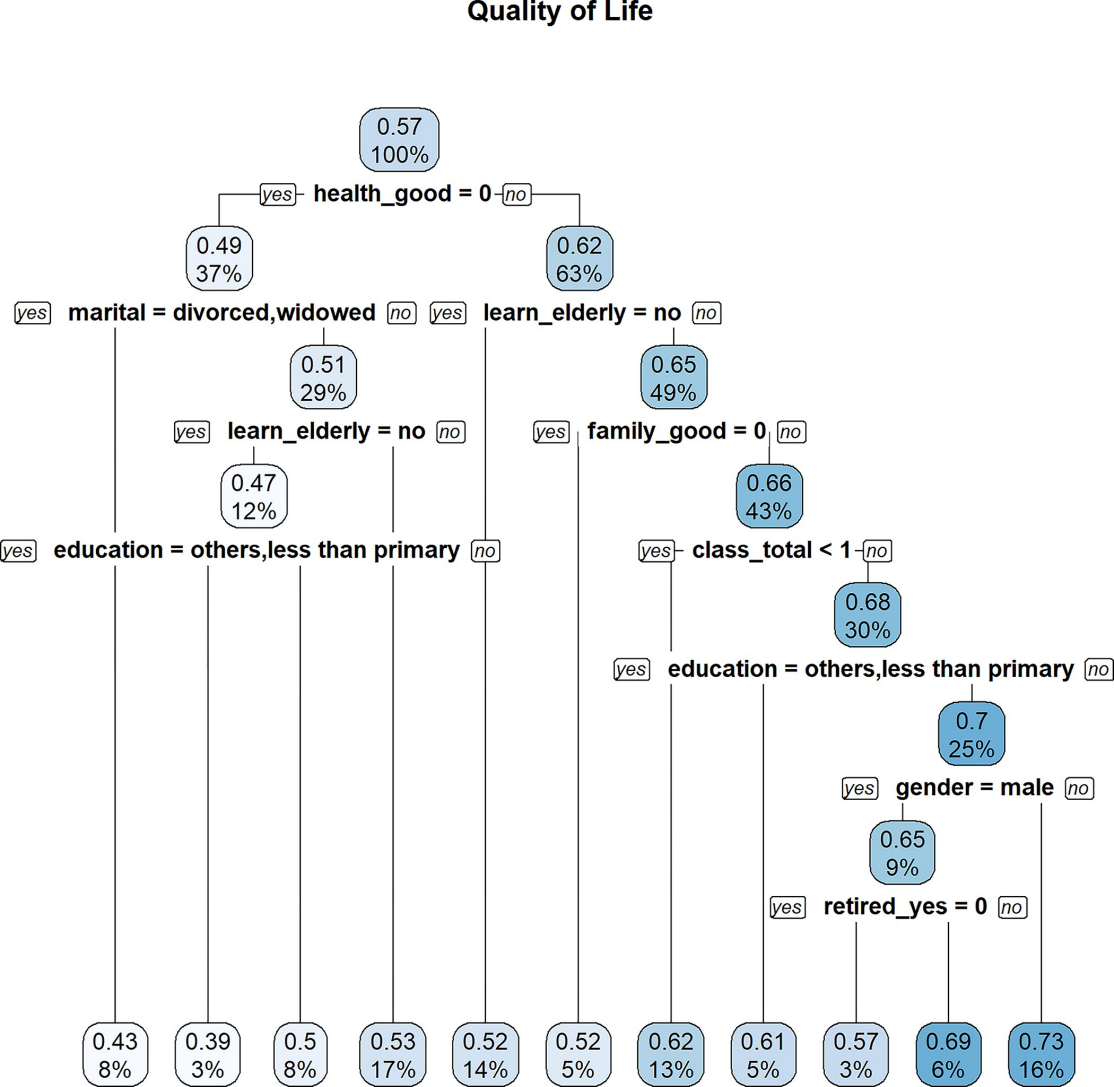
MWI (quality of life) as the outcome variable.

**Fig 10:**
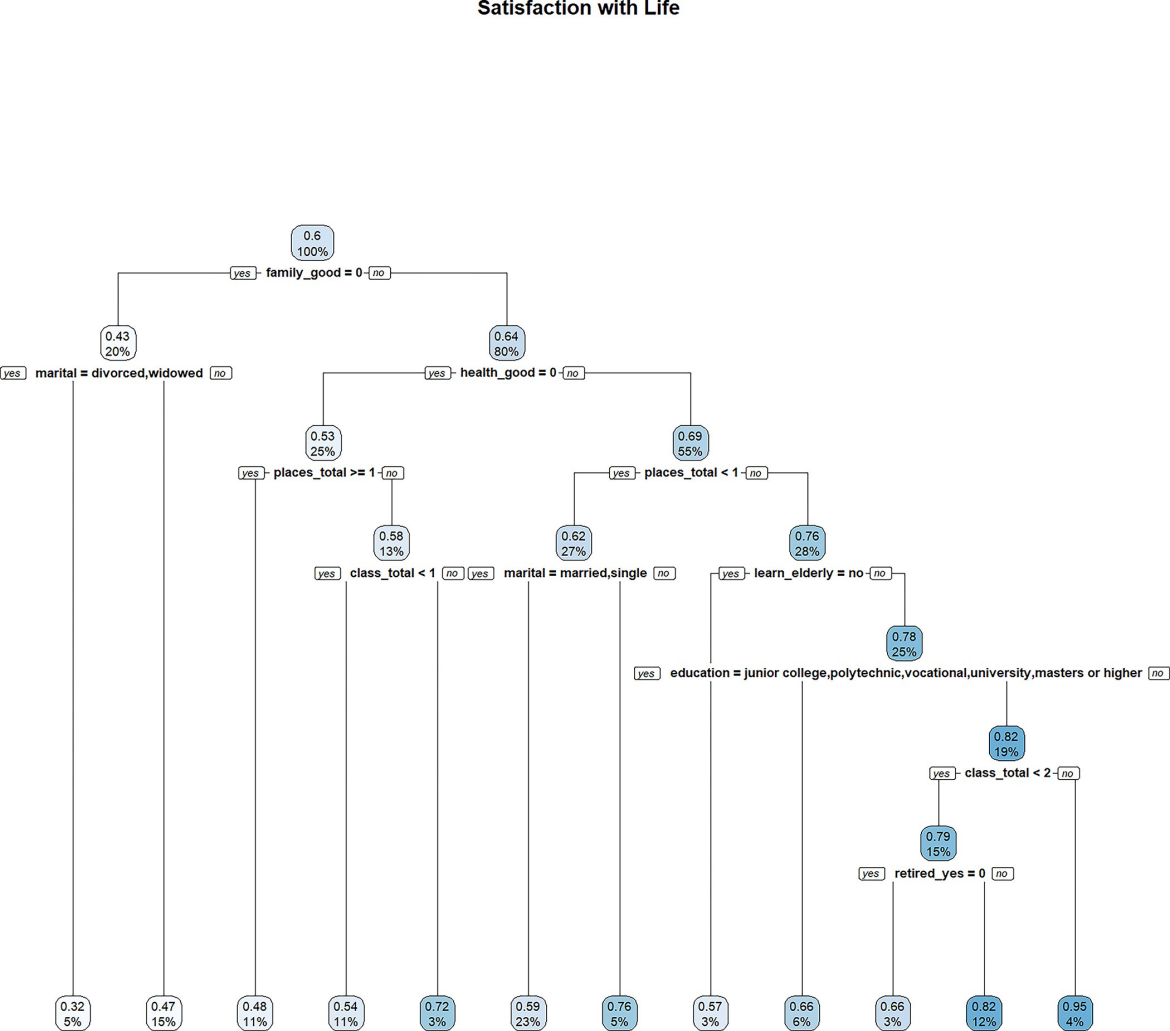
MWI (satisfaction with life) as the outcome variable.

**Fig 11:**
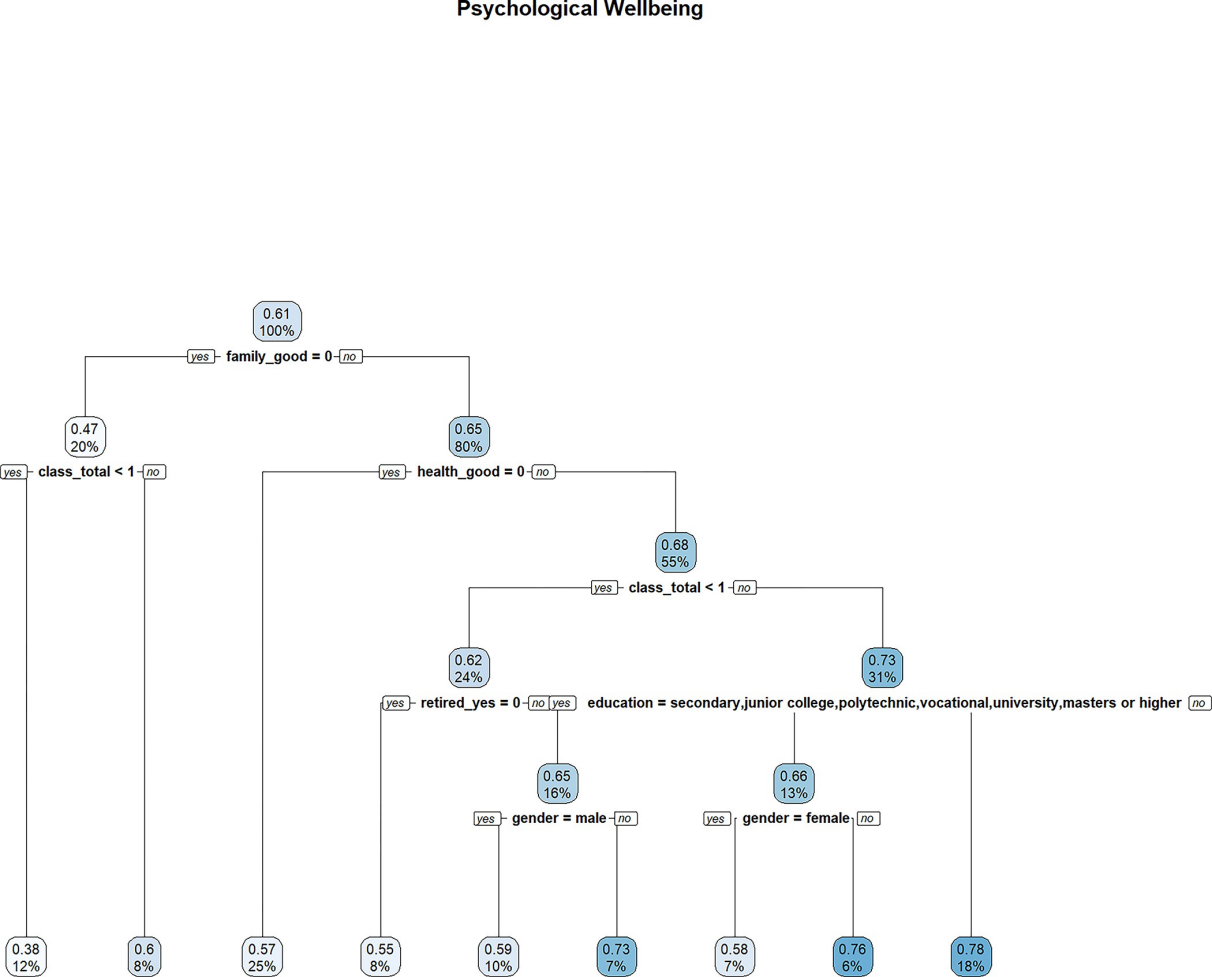
MWI (psychological wellbeing) as the outcome variable.

In Figs 9–11, decision trees are constructed for “Quality of Life”, “Satisfaction with Life”, and “Psychological Wellbeing”, respectively. Each node is labeled with two numbers. The proportion of the sample is reported at the bottom of each node, while the top node shows the average value of the outcome variable based on the sample contained in the node. For example, in Fig 9, the value of 0.57 represents the average MWI score of “Quality of Life” based on the entire sample. The bottom number of the top node is always 100%, indicating that the root node contains the entire sample.

Attributes that are high up in the decision tree are more important, as they determine the initial segmentation of the sample and will influence how the splits are made in the lower part of the tree. Therefore, a good way to assess the importance of lifelong learning for wellbeing, among other attributes, is to see whether a lifelong learning variable splits high up in the tree.

For “Quality of Life”, the first variable used to split the decision tree is *health_good* (i.e., “yes” if the respondent reported having “good” or “very good” health). Thus, good health appears to be important for one’s perceived quality of life. Among those with good health, attitudes towards lifelong learning are the next variable used to split the tree. Therefore, attitudes towards lifelong learning also appear to be important for one’s perceived quality of life.

In the decision tree for “Quality of Life”, the highest average “Quality of Life” score in a leaf node (i.e., the node that terminates at the bottom of the tree) is 0.73. This leaf node comprises 16% of the sample and includes individuals who are healthy, do not have a negative attitude towards lifelong learning (i.e., the negation of the attribute “*learn_elderly_no*”), have taken one or more classes in the past 12 months, have higher than primary education, and are females. Non-retired males with the same profile on health, attitudes towards lifelong learning, and class activities participation have the next highest average “Quality of Life” score. While it is not surprising that health and educational level may affect one’s wellbeing, it is noteworthy to find that lifelong learning is also an important predictor.

Attitudes towards lifelong learning also appear to be relevant for life satisfaction. Fig 10 shows a leaf node with a high MWI score of 0.95 for “Satisfaction with Life”, indicating that individuals with good family relationships (rated as “good” or “excellent”) and good health, who have taken a class activity in the past year, and who do not have a negative attitude towards lifelong learning tend to report higher levels of life satisfaction. By contrast, individuals with similar profiles in family relationships, health, and classes taken, but with a negative attitude towards lifelong learning, exhibit a much lower average MWI score of 0.57, suggesting that negative attitudes towards lifelong learning are associated with lower levels of life satisfaction.

However, attitudes towards lifelong learning does not appear to be important for psychological wellbeing as it is not among its key predictors, as shown in Fig 11. While positive attitudes towards lifelong learning are associated with higher levels of perceived quality of life and life satisfaction, these attitudes do not seem to affect psychological wellbeing in the same way.

While attitudes towards lifelong learning only appear to be relevant for some aspects of wellbeing, the total number of different classes taken in the past 12 months (i.e. *class_total*) appear as a feature in all three decisions trees. However, the total number of different places visited (i.e., *places_total*) only appear in the decision tree for life satisfaction. As participation in classes reflects an engagement in learning, this suggests that learning (not just learning attitude) is associated with general wellbeing.

## 7. Regressions

While decision trees produce results that are easy to understand visually, they do not speak to whether lifelong learning attitudes are statistically significant, let alone their effect size. To complement our decision tree analysis, we estimate a linear regression that relates “Quality of Life”, “Satisfaction with Life” or “Psychological Wellbeing” to the predictors considered in the decision trees. It is important to emphasize that our objective is to explore the statistical significance of the predictors, and not to establish causal relationships. Therefore, the results should be interpreted as associative, not causative.

Before we estimate our regressions, we need to convert the categorical variables into numerical variables. Here, the dummy *learn_elderly* is equal to 1 if respondent said that the elderly should keep on learning (for our decision trees, *learn_elderly* is a “yes” or “no” nominal variable). The variables *class_total* and *activities_total* are the total number of classes or places visited in the last 12 months by the respondent, respectively.

The dummy *health_good* is equal to 1 if the respondent reported to have “good” or “very good” health. The dummies *family_good* and *friends_good* are equal to 1 if the respondent reported to have “good” or “excellent” relationships with family and friends, respectively. The dummies *retired*, *female*, *married* and *secondary_more* indicate if the respondent is retired, a female, married, and has more than secondary level education, respectively. Finally, *no_children* is the number of children that the respondent reported to have (see Table A3 of the S1 Appendix for the variable definitions).

### 7.1 Results

Table 3 reports the regression results with robust standard errors. The variable *learn_elderly* is found to be statistically significant at the 1% level for quality of life, indicating that individuals with positive attitudes towards lifelong learning tend to have a better quality of life. However, *learn_elderly* is not found to be statistically significant for life satisfaction and psychological wellbeing. This finding is consistent with our decision trees, which also suggest that attitudes towards lifelong learning matter for quality of life but not for psychological wellbeing. However, unlike our decision trees, there is no strong evidence to suggest that attitudes towards lifelong learning are associated with life satisfaction.

**Table 3:** Determinants of wellbeing.

	(1)	(2)	(3)
	Quality of Life	Satisfaction with Life	Psychological Wellbeing
*learn_elderly*	0.0611***	0.0414	0.0289
	(0.0186)	(0.0272)	(0.0314)
*class_total*	0.0491***	0.0739***	0.0802***
	(0.0132)	(0.0199)	(0.0231)
*places_total*	0.00355	-0.00305	0.000605
	(0.0136)	(0.0196)	(0.0219)
*disability*	-0.00879	0.0989	0.0137
	(0.0589)	(0.102)	(0.0655)
*mobility*	-0.0197	-0.0601*	-0.0501
	(0.0211)	(0.0350)	(0.0364)
*health_good*	0.0957***	0.108***	0.0972***
	(0.0156)	(0.0250)	(0.0270)
*family_good*	0.0482**	0.128***	0.111***
	(0.0205)	(0.0302)	(0.0334)
*friends_good*	0.0299	0.0281	0.0103
	(0.0224)	(0.0351)	(0.0371)
*retired_yes*	0.0160	0.0844***	0.0658**
	(0.0200)	(0.0280)	(0.0284)
*female*	-0.0259	-0.00938	0.000197
	(0.0172)	(0.0252)	(0.0272)
*married*	0.0260	0.0202	0.0322
	(0.0180)	(0.0271)	(0.0281)
*secondary_more*	0.0173	-0.0620**	-0.0485*
	(0.0188)	(0.0258)	(0.0286)
*no_children*	0.00714	0.00244	-0.000965
	(0.00486)	(0.00712)	(0.00755)
*Constant*	0.343***	0.293***	0.348***
	(0.0294)	(0.0412)	(0.0492)
Observations	292	292	292
R-squared	0.319	0.327	0.259

Robust standard errors in parentheses

*** p<0.01

** p<0.05

* p<0.1

By contrast, participation in class activities is statistically significant for all three aspects of wellbeing. This echoes our findings from the decision trees, where participation in classes are featured in all of them. As foreshadowed by the decision trees, the number of places visited (i.e., *places_total*) is statistically insignificant for all three wellbeing measures. This is not surprising, as we might expect visits to places to be less strongly linked to lifelong learning compared to participation in class activities.

The remaining results from our regressions are similar to what we have found from the decision trees. For instance, the regressions show that good health and family relationships are statistically significant predictors of wellbeing. However, good relationships with friends do not appear to matter. Retirement is statistically significant for life satisfaction and psychological wellbeing, but not for the quality of life. This may be due to the fact that retirement can have both positive and negative effects on wellbeing, and its impact may vary depending on individual circumstances.

Interestingly, educational attainment has a negative association with life satisfaction and psychological wellbeing, suggesting that older adults with higher levels of education may be more unhappy as they age. This finding warrants further investigation to understand the underlying reasons behind this relationship.

We have identified several variables that are statistically insignificant for wellbeing. Marital status and number of children do not have a significant effect, suggesting that it is not these factors themselves that determine wellbeing, but rather the quality of family relationships. Surprisingly, disability and mobility difficulties are statistically insignificant, although this is consistent with the results from the decision tree where these variables are not featured. We estimate the regressions without learn_elderly, class_total and places_total. While mobility difficulties become weakly statistically significant (i.e., at the 10% or 5% level), disabilities remain statistically insignificant. Therefore, there is no evidence that disability is insignificant because attitudes towards lifelong learning, participation in class activities, and visits to places have driven out its effects. Furthermore, gender is also found to be statistically significant, indicating that a person’s gender does not determine their wellbeing.

## 8. Discussion

This is one of the first studies to explore empirically whether lifelong learning attitudes are associated with the elderly subjective wellbeing. This issue is important from the Singapore policymakers’ perspective. In Singapore, continuous learning is encouraged for people of all ages. For example, Singapore citizens and permanent residents below the age of 40 are given 70% subsidies on fees of courses identified by the Singapore SkillsFuture SG (SSG) agency that meet its skills framework, while those aged 40 and above receive 90% of course fee subsidy. These courses cover a wide range of areas, which include the training of practitioners like the training of a real estate salesperson to more technological-focused training in areas such as data science and blockchain finance (see https://www.myskillsfuture.gov.sg/content/portal/en/training-exchange/course-landing.html for a list of courses).

Besides providing skills training, continuous education is also encouraged for another reason. According to the Singapore Civil Service College, “Singapore wants to develop a culture of lifelong learning, where people get satisfaction in life from learning at every stage regardless of where they start” (see https://knowledge.csc.gov.sg/digital-issue-10/lifelong-learning-through-a-global-lens/). Additionally, the Singapore Ministry of Health has a policy initiative for successful ageing, called the “I Feel Young SG” action plan, that encourages lifelong learning among seniors. To do so, the government has set up a National Silver Academy “to provide a wide range of learning opportunities for seniors to learn for interest and stay active”. While it is believed that seniors engaging in learning would have better well-being, self-confidence, life satisfaction and self-efficacy [25, 27], in the context of Singapore, there is little empirical evidence to suggest that lifelong learning attitudes are associated with the elderly wellbeing. The objective of this study is to explore if such an association is present in Singapore.

In general, there is a dearth of international evidence, let alone evidence based on Singapore. One study related to our paper is [30], which focused on understanding the characteristics and motivations of older Singaporeans who are engaged in learning with the National Silver Academy, and whether there are personal and structural barriers to continuation of learning. However, their work differs from ours in that it is largely exploratory, whereas our paper attempts to distill evidence on associations using machine learning approaches. Another study related to our paper is [29], a research commissioned by the Singapore government-linked agency, Council of Third Age (C3A). Unlike our paper, which focuses on seniors aged 65 and older, this study employed a smaller sample of adults aged 50 to 64 years. Moreover, our study follows a quantitative approach, while [29] followed a qualitative approach, which provides limited evidence on the benefits of lifelong learning. Our paper is the first quantitative study that combines primary data collection and machine learning approach to rigorously study the association between learning and wellbeing among seniors in Singapore.

Besides our study and [29, 30], much of the research on the impact of learning by older adults comes from North America and Europe. For example, [23] found that continuous learning positively affects the physical, psychological, and social health of older adult participants in the US. For the UK, [24] found that participation in lifelong learning had positive effects upon a range of health outcomes including mental health resilience and one’s ability to cope with stress. Similarly, [41] presented evidence on the wider benefits of learning, such as one’s psychological wellbeing. Overall, these studies suggest that there are benefits of learning for seniors in terms of life satisfaction and improved psychosocial outcomes.

## 9. Conclusion

We conducted a primary survey on 300 respondents to study if there is an association between lifelong learning and subjective wellbeing of those aged 65 and above in Singapore. We construct a novel index to measure three different aspects of wellbeing (quality of life, life satisfaction, and psychological health) and explore if wellbeing is associated with attitudes towards lifelong learning using machine learning approaches.

Our analyses indicate that attitudes towards lifelong learning play an important role in shaping the elderly’s wellbeing, particularly their quality of life. Moreover, participation in class activities, which we interpret as form of light learning engagement, is important for all three aspects of wellbeing. Overall, our findings provide support for the idea that promoting lifelong learning among older adults, especially in the form of classes, can have positive impacts on their overall wellbeing.

As a caveat, it is important to emphasize that our study only focuses on associative, not causative relationships. Our findings are based on a specific sample of elderly individuals in Singapore and may not be generalizable to other populations. Nonetheless, our results provide a starting point for policy discussions and highlight the need for further research in this area.

Our paper provides foundational evidence on the potential association between elderly wellbeing and their attitudes towards lifelong learning. For future work, we may extend our research in the following ways. First, we may build upon the existing work by conducting a larger scale survey and introduce interventions to identify possible causal relationship between lifelong learning behaviours and improvements in subjective wellbeing among the elderly. Second, to assess the external validity of our study, we may extend our analysis to other countries and cultures to check how robust the association between lifelong learning and elderly wellbeing is and whether this association could be influence by cross-cultural and institutional differences. Finally, we may attempt to delve deeper into mechanisms that explain why lifelong learning affects the subjective wellbeing among the elderly. Detailed investigation on the forms of learning, and potential mediators or moderators (such as health status, social support, and cognitive abilities), complemented by the qualitative analysis such as interviews and focus group discussion, could provide insightful knowledge on the underlying mechanism. Thereafter, evidence-based policy recommendations can be formulated to integrate learning programmes into elderly care policies to enhance their subjective wellbeing.

## Supporting information

S1 Appendix (DOCX)

S1 Dataset Survey responses from 300 respondents. (ZIP)
