# Supplementary material for: Does lifelong learning matter for the subjective wellbeing of the elderly? A machine learning analysis on Singapore data
Source: PLoS One. 2024 Jun 5;19(6):e0303478. doi: 10.1371/journal.pone.0303478 (PMC11152262; doi:10.1371/journal.pone.0303478)
Supplement: S1 Appendix — (DOCX) [file pone.0303478.s001.docx]

# Appendix 1

**Table A1: Survey Questions on the Wellbeing**

1. **Survey questions on the “Quality of Life”**

Please indicate how frequently in the past week you have experienced the following: (1: Often; 2: Sometimes; 3: Not often; 4: Never)

| 1 | My age prevents me from doing the things I would like to |
| --- | --- |
| 2 | I feel that what happens to me is out of my control |
| 3 | I feel free to plan for the future |
| 4 | I feel left out of things |
| 5 | I can do the things I want to do |
| 6 | Family responsibilities prevent me from doing what I want to do |
| 7 | I feel that I can please myself what I do |
| 8 | My health stops me from doing things I want to |
| 9 | Shortage of money stops me from doing the things I want to do |
| 10 | I look forward to each day |
| 11 | I feel that my life has meaning |
| 12 | I enjoy the things that I do |
| 13 | I enjoy being in the company of others |
| 14 | On balance, I look back on my life with a sense of happiness |
| 15 | I feel full of energy these days |
| 16 | I choose to do things that I have never done before |
| 17 | I am satisfied with the way my life has turned out |
| 18 | I feel that life is full of opportunities |
| 19 | I feel that the future looks good for me |

1. **Survey questions on the “Satisfaction with Life”**

Below are five statements that you may agree or disagree with. Using the 1 - 7 scale (1: Strongly disagree; 2: Disagree; 3: Slightly disagree; 4: Neither agree nor disagree; 5: Slightly agree; 6: Agree; 7: Strongly agree), indicate your agreement with each item by placing the appropriate number in the box after the statement. Please be open and honest in your responding.

| 1 | In most ways my life is close to my ideal |
| --- | --- |
| 2 | The conditions of my life are excellent |
| 3 | I am satisfied with my life |
| 4 | So far I have gotten the important things I want in life. |
| 5 | If I could live my life over, I would change almost nothing. |

1. **Survey questions on the “Psychological Wellbeing”**

Please indicate for each of the five statements which is closet to how you have been feeling over the past two weeks: (1: All of the time; 2: Most of the time; 3: More than half of the time; 4: Less than half of the time; 5: Some of the time; 6: At no time)

| 1 | I have felt cheerful and in good spirits |
| --- | --- |
| 2 | I have felt calm and relaxed |
| 3 | I have felt active and vigorous |
| 4 | I woke up feeling fresh and rested |
| 5 | My daily life has been filled with things that interest me |

**Table A2**: Definition of Attributes for MCA

| **Variable** | **Definitions** |
| --- | --- |
| *MWI_Quality_r_Q#* | Multidimensional welfare index (MWI) score for “Quality of Life”. The tag _r_Q# indicates if the score is ranked in the # quartile. |
| *MWI_Satisfaction_r_Q#* | Multidimensional welfare index (MWI) score for “Satisfaction with Life”. The tag _r_Q# indicates if the score is ranked in the # quartile. |
| *MWI_Psychological_r_Q#* | Multidimensional welfare index (MWI) score for “Psychological Wellbeing”. The tag _r_Q# indicates if the score is ranked in the # quartile. |
| *learn_elderly* | = “yes” if the respondent said the elderly should keep learning |
| *class_total* | =1 if the respondent had taken two more or different classes, among singing, dancing, music, exercise or others, in the last 12 months |
| *places_total* | =1 if the respondent had been to two or more places, among gym, sporting club, museum, library and an institute of learning, in the last 12 months |
| *mobility* | = “yes” if the respondent has mobility difficulty |
| *disability* | = “yes” if the respondent has a disability |
| *no_children* | = “no child” if the respondent is childless,  = “1-3 children” if the respondent has 1 to 3 children, = “more than 3 children” if the respondent has more than 3 children |
| *health* | = “good” if health is good or very good,  = “not good” if otherwise |
| *family* | = “good” if family relationship is good or excellent, = “not good” if otherwise |
| *friends* | = “good” if relationship with friends is good or excellent, = “not good” if otherwise |
| *working_status* | = “yes” if still working full-time, part-time, ad-hoc or freelance |
| *gender* | = “female” if the respondent is female, = “male” if the respondent is male |
| *marital* | = “married” if the respondent is married, = “not married” if the respondent is single, widowed, or divorced |
| *education* | = “more than secondary” if the respondent has more than secondary level education, = “secondary or less” if otherwise |

**Table A3**: Definition of Variables for Decision Trees and Regressions

| **Variable** | **Definitions** | **Decision Trees^1^** |  | **Regressions^1^** |
| --- | --- | --- | --- | --- |
| *MWI_Quality* | Multidimensional welfare index (MWI) score for “Quality of Life” (0 lowest and 1 highest) | Y |  | Y |
| *MWI_Satisfaction* | Multidimensional welfare index (MWI) score for “Satisfaction with Life” (0 lowest and 1 highest) | Y |  | Y |
| *MWI_Psychological* | Multidimensional welfare index (MWI) score for “Psychological Wellbeing” (0 lowest and 1 highest) |  |  |  |
| *learn_elderly* | = 1 (for regression) or “yes”/“no” (for MCA/decision trees) if the respondent said the elderly should keep learning/do not need to learn | Y |  | Y |
| *class_total* | Total number of different classes, i.e. singing, dancing, music, exercise or others, the respondent has taken in the last 12 months | Y |  | Y |
| *places_total* | Total number of different places, i.e. gym, sporting club, museum, library, institute of learning, or others, the respondent has been to in the last 12 months | Y |  | Y |
| *mobility* | = “yes” (decision trees) or = 1 (regressions) if the respondent has mobility difficulty | Y |  | Y |
| *disability* | = “yes” (decision trees) or = 1 (regressions) if the respondent has a disability | Y |  | Y |
| *no_children* | Number of children |  |  | Y |
| *children_0* | = 1 if number of children is 0 | Y |  |  |
| *children_13* | = 1 if the number of children is between 1 to 3 | Y |  |  |
| *health_good* | =1 if health is good or very good | Y |  | Y |
| *family_good* | =1 if family relationship is good or excellent | Y |  | Y |
| *friends_good* | =1 if relationship with friends is good or excellent | Y |  | Y |
| *retired_yes* | =1 if not working | Y |  | Y |
| *female* | =1 if female | Y |  | Y |
| *married* | =1 if married | Y |  | Y |
| *education* | primary, secondary, junior college, polytechnic, university, masters or higher | Y |  |  |
| *secondary_more* | =1 if education level is more than secondary school |  |  | Y |

**^1^**The columns show the variables used in our decision trees or regressions.
